# Phase separation in gene transcription control

**DOI:** 10.3724/abbs.2023099

**Published:** 2023-06-01

**Authors:** Chengyu Li, Zhuo Li, Zhibing Wu, Huasong Lu

**Affiliations:** 1 Zhejiang Provincial Key Laboratory for Cancer Molecular Cell Biology Life Sciences Institute Zhejiang University Hangzhou 310058 China; 2 Department of Oncology Affiliated Zhejiang Hospital Zhejiang University School of Medicine Hangzhou 310058 China

**Keywords:** RNA Pol II, transcriptional condensate, phase separation

## Abstract

Phase separation provides a general mechanism for the formation of biomolecular condensates, and it plays a vital role in regulating diverse cellular processes, including gene expression. Although the role of transcription factors and coactivators in regulating transcription has long been understood, how phase separation is involved in this process is just beginning to be explored. In this review, we highlight recent advance in elucidating the molecular mechanisms and functions of transcriptional condensates in gene expression control. We discuss the different condensates formed at each stage of the transcription cycle and how they are dynamically regulated in response to diverse cellular and extracellular cues that cause rapid changes in gene expression. Furthermore, we present new findings regarding the dysregulation of transcription condensates and their implications in human diseases.

## Introduction

The central dogma of molecular biology, initially stated by Francis Crick [1], establishes the framework for understanding how the genetic information embedded in DNA is decoded in living organisms. Transcription is one of the key steps in the central dogma that provides a bridge between DNA and the functional gene product. As such, it has been recognized as a command-and-control center to achieve specific gene expression patterns critical for virtually all aspects of cellular function. In eukaryotic cells, transcription is carried out by the multi-subunit RNA polymerase (Pol) enzyme, of which three different types have been isolated, including Pol I, Pol II, and Pol III [2]. Among these polymerases, Pol I synthesizes the pre-ribosomal RNA (rRNA), Pol II produces the precursors of messenger RNA (mRNA) and a variety of non-coding RNA, and Pol III is responsible for the transcription of transfer RNAs (tRNA), rRNA 5S and other small RNAs [3]. In this review article, we primarily focus on Pol II transcription; readers interested in Pol I and Pol III transcription are referred to several excellent reviews for detailed discussion [ 4– 6] .

Pol II transcription is a complex multi-step process. It starts with the assembly of a preinitiation complex (PIC) composed of Pol II, general transcription factors (GTFs) and the Mediator complex at a gene promoter region, where the two strands of DNA in the PIC are denatured to allow the template strand being positioned at the Pol II active center to initiate the RNA synthesis. Once the length of nascent RNA reaches to a critical length, Pol II escapes from the promoter region and forms an elongation complex to further extends the RNA chain until it travels to the end of the gene and terminates the transcription cycle [ 7– 9] . Over the past decades, each of these transcription steps has been studied in great depth, revealing a plethora of transcription factors and co-regulators that enable the fine regulation of Poll II transcription.

Although much progress has been made in elucidating the key events during transcription, what is often overlooked is the fact that transcription takes place strictly in a spatially-defined manner [3]. However, we still lack complete knowledge of how this spatial organization of transcription is achieved in the nucleus. Interest in this aspect has recently surged due to the discoveries that condensation by certain transcription-related factors is a widespread phenomenon, provoking rampant speculations on a phase separation model for transcriptional control.

In this review, we focus on the molecular mechanisms and functions of nuclear condensates in transcriptional regulation. We first summarize the condensates identified at different stages of the transcription cycle, followed by a discussion on the factors and pathways controlling the interplays between these separate condensates and how they are dynamically regulated in response to diverse cellular and extracellular cues that cause rapid changes in gene expression. Finally, we present new findings regarding the dysregulation of transcription condensates and their roles in human diseases.

## Formation of Biomolecular Condensates via Phase Separation

The biomolecules, including proteins and nucleic acids, must be strictly compartmentalized to elicit their corresponding functions at the correct time and space in cells. While forming various membrane-encapsulated organelles offers a means to compartmentalize and concentrate specific sets of molecules at discrete intracellular locations, many cellular compartments that are not bound by membranes also constitute an important mechanism to achieve spatiotemporal regulation of biological reactions [ 10, 11] . Despite the absence of a physical membrane to separate their contents from the surrounding environment, these structures are all characterized by their ability to locally concentrate biological molecules, raising a long-lasting question about their formation.

**Figure 1:**
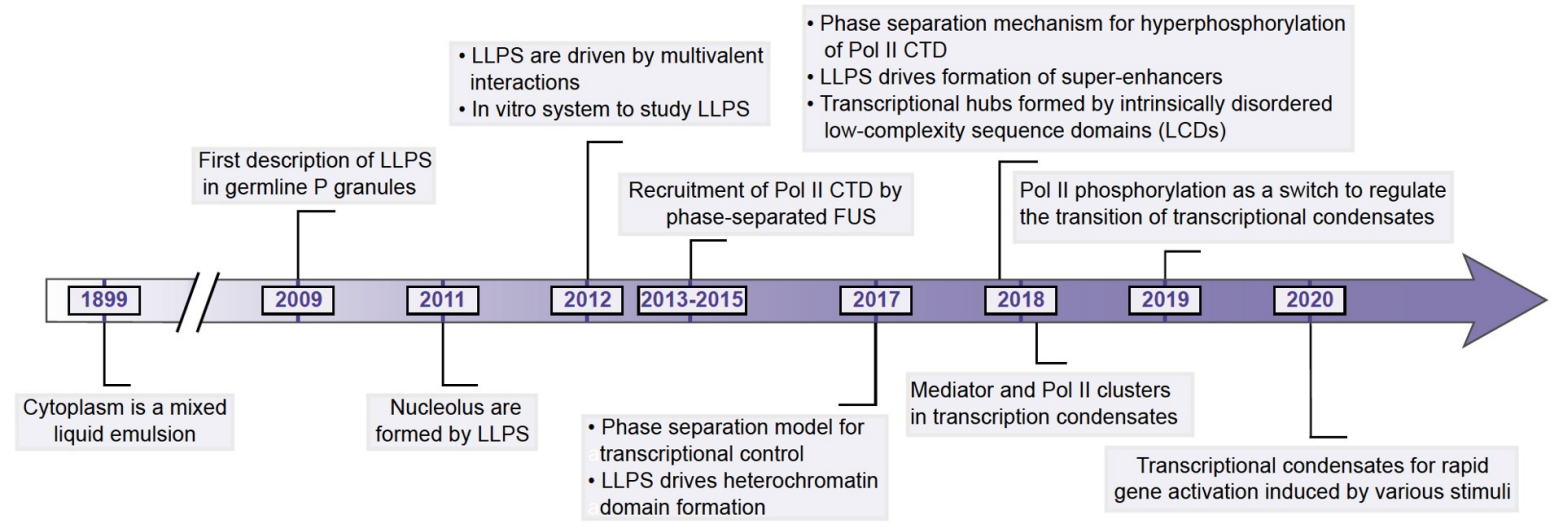
Figure 1 Timeline of key discoveries in the field of phase separation and transcriptional control

Research into this question has revealed that these membraneless compartments, also called biomolecular condensates, are formed in part through phase separation, a physiochemical phenomenon that refers to the de-mixing process that forms dense and dilute phases from a homogeneous solution [ 10, 12] . The chemical potential in both phases becomes equal through phase separation, allowing the biomolecular condensates to be maintained in an energetically favorable state without constant energy input. In the past decades, progress in deciphering the mechanism and function of biomolecular condensates has been extremely rapid ( Figure 1). It has now become increasingly clear that biomolecular condensates play a pivotal role in different cellular processes, such as signal transduction, stress response, and transcription regulation, and their dysfunction is closely related to the occurrence of many human diseases.

Experimental evidence demonstrates that multivalent interactions are molecular driving forces underlying phase separation. Within the biomolecular condensates, multivalent molecules function as the scaffold to enable the assembly of phase-separated structures, whereas client components are often at a lower valency and are recruited to the condensates by directly binding to scaffolds [ 10, 13, 14] . In the case of protein phase separation, two types of multivalent interactions have been reported to drive the assembly of biomolecular condensates. The first one relies on the modular interaction domains, which are folded by evolutionarily conserved protein segments. When multiple of these domains are present in one protein, they are expected to create multivalency that enables the engagement of multiple binding partners and the formation of dynamic interacting networks to promote phase separation, reminiscent of classic principles in polymer science [ 13, 15] . Many natural or engineered proteins composed of repeated modular domains have been shown to undergo phase separation and lead to the formation of biomolecular condensates [ 13, 14] . In addition, many protein domains such as the coiled-coil domain that mediates hydrophobic interactions have also been identified as key regions participating in protein phase separation [ 16– 19] . Another type of multivalent interactions is mediated by proteins containing long stretches of intrinsically disordered regions (IDRs). Although IDRs lack a defined 3D structure, they often have low sequence complexity and contain repeated sequence elements such as tyrosine, glycine and serine, providing the multivalency to enable various homotypic and heterotypic interactions [ 20, 21] . Not surprisingly, many intracellular condensates are highly enriched with IDR-containing proteins [ 22– 25] . It is worth mentioning that many transcriptional activation domains are comprised of low-complexity (LC) sequences that exist in an intrinsically disordered conformation. For example, the LC domain of the RNA-binding protein FUS can undergo phase separation and form fibrous polymers, which has been reported to directly bind the C-terminal domain (CTD) of Pol II and function as transcriptional activation domains [ 25, 26] . These pioneering studies have added an important new dimension to our knowledge of the mechanistic basis for IDR-mediated gene activation and laid the early foundation for the recent outbreak of the phase separation model for transcriptional control.

## The Spatial Control of Transcription in Cells

Even before the era of phase separation, it has long been known that transcription occurs in a compartmentalized manner inside the nucleus. The nucleolus, for example, is a subnuclear compartment where ribosomal RNA genes are transcribed, processed, and assembled into ribosomal ribonucleoprotein complexes [27]. As for Pol II transcription, a model called “transcription factories” has been proposed to explain how the coordination of numerous transcription-related factors is accomplished in the nucleus. Unlike the conventional view describes the transcription proceeding in a fashion that active transcribing Pol II tracks along its template to synthesize the nascent RNA, with the template DNA being considered relatively static, the transcription factory model argues that it is the gene loci that slide into the specialized location of the factory where transcription machinery and many regulatory transcription factors are enriched to efficiently promote gene transcription [28]. In support of this model, fluorescent labeling of nascent RNA revealed that they are synthesized in discrete and confined domains, therefore providing clues for the scattering distribution of Pol II in the nucleus [ 29– 31] . Furthermore, using super-resolution microscopy combined with live cell imaging techniques, researchers were able to directly visualize the clustering of Pol II in the nucleus [ 32, 33] . While these studies have demonstrated the existence of discrete transcription domains in cells, many aspects of the transcription factories model, particularly the driving forces that initiate their assembly, are not yet fully understood.

## Formation of Biomolecular Condensates at Different Stages of Transcription Cycle

**Figure 2:**
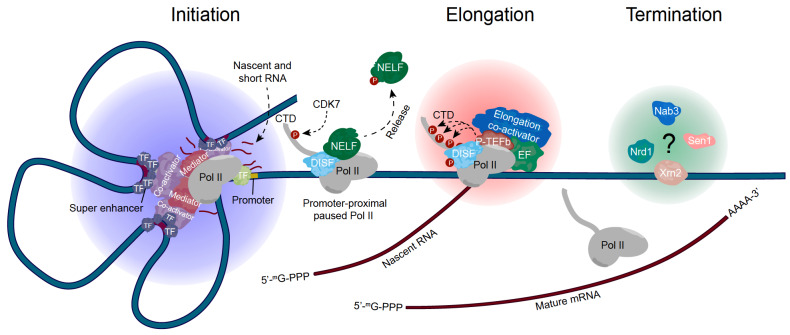
Figure 2 Basal transcriptional condensates for transcriptional control During transcription initiation, RNA polymerase (Pol) II, transcription factors, and co-activators are recruited to super enhancers, where they form liquid-like condensates to promote transcription initiation. As the transcription process continues, these initiation condensates dissolve, and elongation condensates form to facilitate the hyperphosphorylation of the C-terminal domain of RPB1 in Pol II and robust transcription elongation. Finally, at the termination stage, the mature RNA is released from Pol II, and the transcription complex dissociates. Whether or not phase separation is involved in this process remains to be documented.

Accurate transcription requires the dynamic assembly of transcription machinery and myriads of regulatory factors at specific times and locations. Recent research suggests that functionally distinctive transcriptional condensates enriched with different sets of factors play a key role in regulating this spatial control ( Figure 2). Since these condensates are required for gene transcription under normal conditions, they are referred to as basal transcriptional condensates. In this section, we discuss recent advances in our understanding of these basal transcriptional condensates and their role in gene expression.

### Condensates formed at super-enhancers

Super-enhancers are large clusters of enhancers that play an important role in driving the expression of genes involved in cell identity and development [34]. They are characterized by an extreme enrichment for the binding of master transcription factors and coactivators, which leads to the formation of a compartmentalized transcriptional domain. Within this domain, many cooperative binding events can occur between different regulatory components, driving robust transcriptional activation of target genes. Another prominent feature of super-enhancers is that they are highly dynamic, often exhibiting sharp transitions during their formation and dissolution [ 35, 36] . This dynamic nature of super-enhancers is thought to emanate from a single nucleation event that relies on cooperativity among different regulatory components.

In light of these unique features, it was proposed that the dense assemblies of the transcriptional machinery and regulatory factors at active super-enhancers are formed via phase separation and exist in the form of biomolecular condensates [37]. Consistent with this hypothesis, BRD4 and MED1, two transcriptional coactivators known to reside at super-enhancers, were recently reported to form nuclear puncta in cells [ 38, 39] . MED1 is a subunit of the large transcriptional Mediator complex, which as a whole is composed of approximately 30 subunits. As expected, the entire Mediator complex is found within the puncta at super-enhancers, wherein Pol II clusters are also co-localized in a transcription-dependent manner. Live cell imaging with super-resolution microscopy further revealed that molecules within these puncta could undergo dynamic and rapid exchange with their surrounding environment, consistent with properties of biomolecular condensates formed by phase separation [39]. Sequence analysis of BRD4 and MED1 revealed that both proteins harbor long stretch of IDRs, which were shown to facilitate condensates formation at active super-enhancers. This suggests that these IDRs are important for condensate formation and transcriptional regulation in cells. Consistent with this notion, reconstituted MED1-IDR droplets are capable of depleting transcription apparatus from nuclear extracts. In addition, a number of transcriptional co-activators, including Brd4, OCT4, GCN4, and YY1, have been reported to form phase-separated condensates at enhancers, promoting specific gene expression programs crucial for cell fate determination [ 38, 40– 46] . Recent study also reported that enhancer RNAs can recruit nuclear m6A reader YTHDC1, which facilitates the formation of condensates for gene activation [47]. Thus, the formation of biomolecular condensates at super-enhancers offers a strategy to recruit more factors and regulate gene expression more effectively than what would be possible based solely on their sequence nature.

### Condensates formed at transcription initiation stage

As the first step of gene transcription, transcription initiation is a complex process that involves the formation of a pre-initiation complex and the unwinding of the DNA double helix. Among the many proteins known to participate in the initiation process, Pol II lies at the core position because it serves as the scaffold for the pre-initiation complex, allowing other factors to interact with it and coordinate the initiation of transcription. This scaffolding ability of Pol II arises from its CTD, which is a tail-like extension from the body of Pol II [48]. The structure of CTD is flexible and highly disordered, comprising 52 heptad repeats with the consensus sequence YSPTSPS in human cells. These unique characteristics make CTD well-suited for driving the formation of phase-separated condensates.

Accumulating evidence suggests a close link between CTD and phase separation in promoting efficient transcription initiation. On one hand, CTD has been suggested to facilitate phase separation through interactions between CTDs and with other activators [49]. Its multiple binding sites for other proteins allow for the enrichment of a variety of transcription regulators in CTD-containing condensates, creating a reservoir of transcription apparatus to enable efficient initiation at active genes. On the other hand, CTD may also act as a client, being recruited into transcription hubs formed by specific transcription factors. For example, hydrogels assembled by the LCDs of FUS and TAF15 were found to bind directly with and trap CTD to increase the transcriptional initiation of target genes [ 25, 26] . Notably, this favorable partition of CTD into LCD condensates is reversibly controlled in a manner dependent on CTD phosphorylation [ 25, 49] , which provides an additional layer of regulation for Pol II during a transcription cycle. Furthermore, activation domains from a handful of transcription factors can form phase-separated condensates with coactivators, a process in which the CTD is likely to play a role in activating gene expression [41]. Together, these studies revealed the crucial role of CTD in phase separation during transcription initiation. However, many issues, such as the structural basis as well as the functional importance of this regulation, are yet to be determined.

### Condensates formed at transcription elongation stage

After escaping the promoter, Pol II does not immediately advance into the productive elongation phase to synthesize the full-length transcript. Instead, it is initially paused, where a significant portion of Pol II molecules either stop forward translocation or turn to premature termination. This can be caused by a variety of factors, including intrinsic regulatory mechanisms and extrinsic obstacles, such as the presence of inhibitory molecules or physical barriers on the template DNA [50]. The pause of Pol II near the promoter-proximal region is particularly common for genes involved in development and stress response, which is established by the negative elongation factor (NELF) and DRB sensitivity-inducing factor (DSIF) [51]. In contrast, the subsequent release of paused Pol II is triggered by the positive transcription elongation factor b (P-TEFb), a protein kinase complex comprising cyclin-dependent kinase CDK9 and CycT1 [52]. To convert Pol II from promoter-proximal pausing into the productive elongation phase, CDK9 phosphorylates DSIF, NELF, and Ser2 residue of Pol II CTD. Upon phosphorylation, the inhibitory effects of DSIF and NELF on paused Pol II are removed, while the phosphorylated CTD is transformed into a docking platform to recruit many cofactors to stimulate Pol II elongation and co-transcriptional RNA processing [ 8, 53] . Thus, P-TEFb functions as a master regulator for transcriptional elongation control.

Several recent studies have highlighted the importance of phase separation in regulating Pol II transcription elongation. As a cyclin partner required for the catalytic activity of CDK9, CycT1 tightly associates with CDK9 through its N-terminal region that is well-folded into structural domain. However, CycT1 also contains an extended C-terminal region that is largely unstructured and intrinsically disordered, with its precise role in regulating P-TEFb activity being unknown for a long time. Recently, it was reported that CycT1 plays a crucial role in driving the assembly of transcription factors-enriched condensates for transcriptional elongation control [54]. The ability of CycT1 to undergo phase separation stems from a histidine-rich domain located within its IDR, which has been shown to concentrate and compartmentalize P-TEFb, Pol II, and possibly other transcription factors into a phase-separated environment for hyperphosphorylation of the Pol II CTD and robust transcriptional elongation.

Inside the cells, P-TEFb activity is also regulated by a complex network of factors that associate with it and regulate its activity in response to different stimuli and cellular conditions. It is known that the majority of P-TEFb is sequestered in the 7SK snRNP, in which the CDK9 kinase activity is inhibited by HEXIM1 to prevent its activation. In addition to residing in the 7SK snRNP, P-TEFb also exists in either the BRD4-containing complex or the super elongation complex (SEC), wherein the P-TEFb is catalytically active to stimulate Pol II elongation. The SEC represents one of the most active P-TEFb-containing complexes, which comprises Pol II elongation factors eleven-nineteen Lys-rich leukemia (ELL) proteins, P-TEFb, and several frequent mixed lineage leukemia (MLL) translocation partners [55]. Like CycT1, SEC subunits ENL and AFF4 also contain IDRs and can form phase-separated condensates enriched with other SEC subunits [56]. The formation of SEC droplets was found to play an important role in rapid gene induction. In cells undergoing serum starvation and re-stimulation, SEC condensates are rapidly assembled in the vicinity of immediate early genes, facilitating their rapid transcriptional induction. Notably, mutations in the SEC subunit, such as the fusion of ENL with MLL, can increase the phase separation of SEC and potentially lead to aberrant transcriptional activation of its target genes. However, the exact mechanisms by which the mutations affect SEC phase separation and contribute to disease pathogenesis are still not fully understood and warrant further investigations.

### Condensates formed at the end of the transcription cycle and beyond

Emerging evidence also revealed a fundamental role of phase separation in regulating the transcriptional events occurred at the end of a transcription cycle. In Arabidopsis, FCA is an RNA-binding protein associated with RNA 3’-end processing factors. A recent study has shown that FCA forms phase-separated nuclear bodies with the help of the coiled-coil protein FLL2 [18]. Through this process, FCA nuclear bodies can concentrate various 3′-end processing components to promote RNA polyadenylation at specific sites.

In addition to the transcriptional condensates described above, many nuclear condensates or membraneless organelles are also involved in gene expression control. For instance, a slew of studies has shown that condensation of key heterochromatin-associated factors, such as HP1 and MeCP2, underpins the formation of heterochromatin and transcriptional silencing [ 57‒ 60] . Detailed discussions on these topics have recently been reviewed elsewhere [ 61, 62] .

## Mechanisms Underlying the Transition of Condensates at Different Stages of Transcription

It has become increasingly clear that Pol II can be incorporated into transcriptional condensates with different components and, correspondingly, can have distinct functions depending on the specific components that are present. However, transcription is also known as a dynamic process that involves the coordination of multiple different phases. Hence, it raises an important question of how Pol II is moved between different condensates as transcription progresses from one stage to another.

Emerging evidence suggests that phosphorylation of Pol II CTD has a switch-like function in determining the partitioning of Pol II into different condensates. During the initiation of transcription at Pol II promoters, the assembly of the pre-initiation complex begins with the recruitment of Pol II containing an unphosphorylated CTD [ 53, 63] . Recent studies have revealed that this unphosphorylated CTD can not only form transcriptional condensate through phase separation, but it can also be incorporated and concentrated into mediator condensates to promote transcription initiation [ 49, 64] . During transcription, the CTD is sequentially phosphorylated by CDK7 in TFIIH and CDK9 in P-TEFb. This cooperative phosphorylation of CTD stimulates the transition of Pol II into the productive elongation stage [ 8, 65] . As the CTD undergoes these changes in phosphorylation, it dissociates from mediator condensates and incorporates into splicing condensates for RNA processing [64]. While these results have established the essential role of CTD phosphorylation in orchestrating the condensate-partitioning behavior of Pol II, further investigations, particularly structural studies, are needed to elucidate the detailed mechanisms governing the dynamic behavior of these phospho-specific CTD condensates during transcription.

In addition to CTD phosphorylation, RNA has also been suggested to play a role in regulating the formation of condensates during transcription. For example, the enhancer and promoter-associated RNAs produced in the early stages of transcription were recently proposed to facilitate transcriptional condensate formation [66]. In this case, the RNAs are negatively charged scaffolds bound by positive charges in the proteins. Although this stimulatory effect of RNA on condensate formation is considered to be predominately driven by the electrostatic interactions between RNAs and IDRs of transcription factors and coactivators, additional factors, such as the RNA-binding proteins (RBPs), may also play a role in this process. Supporting this notion, recent studies showed that RBPs are widely present on chromatins in actively transcribed regions, and they can coordinate with the nascent transcript to promote the formation of transcriptional condensates for gene expression [ 67– 69] .

On the other hand, high levels of RNA produced from gene transcription can provide negative feedback control on transcriptional condensate formation. Transcription is well-known to be stochastic in nature, switching between active and inactive states periodically [70]. As such, it is conceivable that a high local concentration of RNAs synthesized from a burst of elongation may alter the charge balance between RNA and proteins and lead to the disruption of condensate, as observed in many other cases [ 71– 73] . Indeed, inhibition of transcription elongation leads to decreased RNA synthesis and increased condensate size and lifetime, whereas artificially increasing the level of local RNA interferes with condensate formation and transcription. Thus, RNA molecules can play multifaceted roles in the regulation of transcriptional condensates, providing a feedback mechanism in tuning local gene expression.

## Dynamic Control of Biomolecular Condensates for Transcriptional Regulation

**Figure 3:**
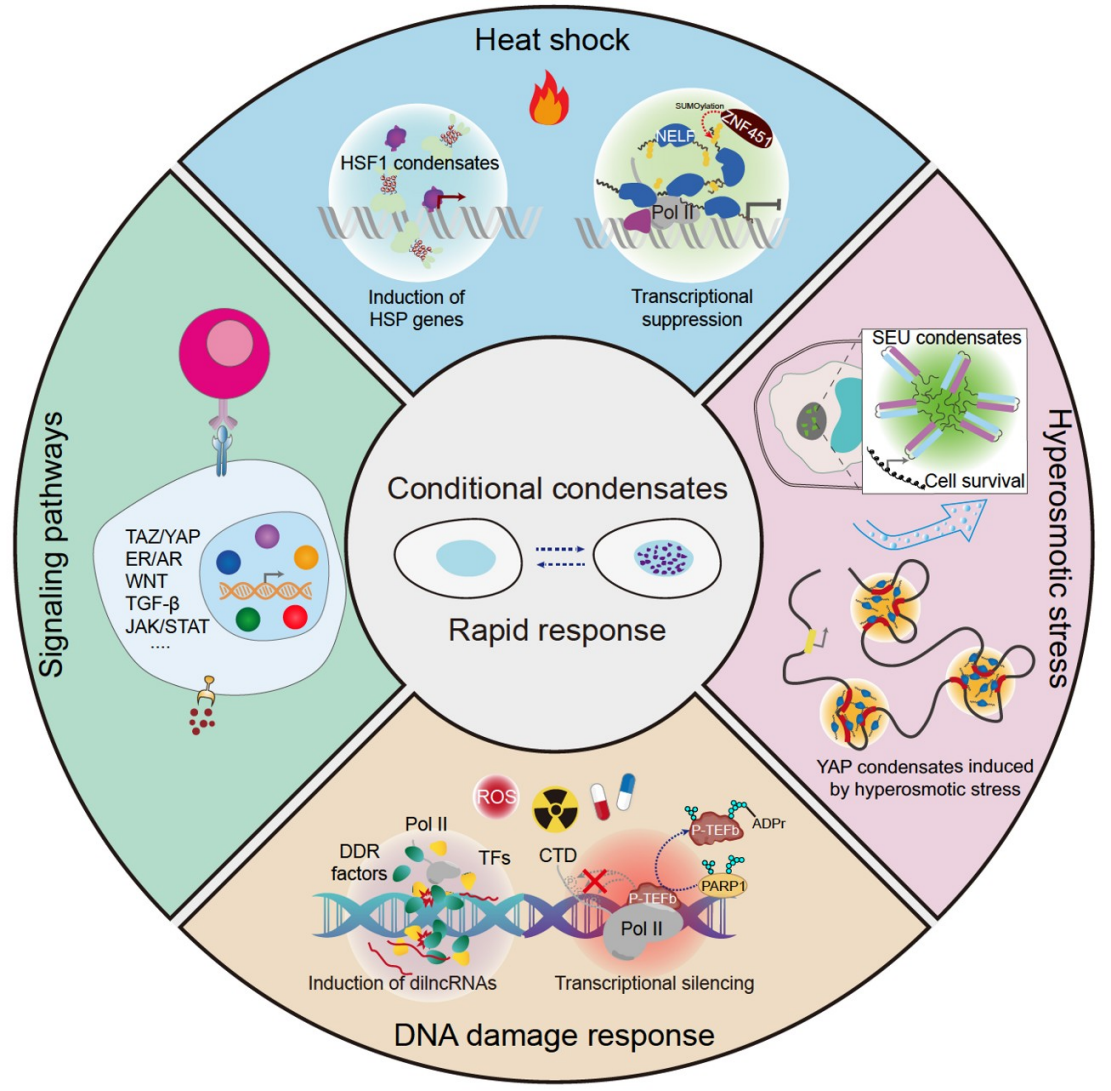
Figure 3 Dynamic control of transcriptional condensates for rapid transcriptional response Conditional transcriptional condensates are regulatory hubs that can rapidly regulate the expression of target genes in response to stress or environmental changes. Under heat shock, both positive (HSF1) and negative (NELF) condensates are quickly formed to orchestrate an efficient and specific gene expression program. In plant cells, the transcription regulator SEU forms phase-separated condensates upon exposure to hyperosmotic stress, which can activate the expression of osmotic-related genes to protect cells from hazards. In mammalian cells, osmotic stress can also trigger the formation of YAP condensates to activate YAP signaling and drive the long-term expression of target genes. After DNA damage, condensates are formed to promote the rapid expression of damage-induced long non-coding RNAs, which in turn facilitate the assembly of DDR foci for efficient DNA repair. However, DNA damage is also known to cause global transcriptional inhibition. The PARylation of P-TEFb subunit CycT1 by PARP1 results in the disruption of P-TEFb’s phase separation, preventing CDK9, the kinase subunit of P-TEFb, to hyperphosphorylate Pol II and inhibiting transcriptional elongation. Finally, many signaling molecules and effectors can form biomolecular condensates to promote the efficient, signal-induced expression of target genes.

Transcription factors are classified into general transcription factors and gene-specific transcription factors, which provide ubiquitous and diverse mechanisms for gene regulation, respectively. Likewise, some transcriptional condensates, such as those discussed above, are indispensable for transcription and paly no part in specific gene regulation, whereas others may only engage in the transcription of a specific set of genes. These condensates act as regulatory compartments, turning the expression of their target genes on or off in response to various signaling pathways or environmental cues. Recent advances have provided an unprecedented view of how these conditional transcriptional condensates act in cells to enable rapid gene expression control ( Figure 3).

### Regulation of biomolecular condensates in response to heat shock

In response to heat shock, cells elicit a complex transcriptional change to adapt to stress and prevent damage. This process is mediated by a group of transcription factors known as heat shock factors (HSFs), which upregulates a transcriptional program consisting of chaperone genes to maintain protein homeostasis and support cellular function [ 74, 75] . Recent studies revealed that HSF1 forms dynamic foci at heat-shock-protein (HSP) gene loci after heat shock in living cells, and this process is driven by phase separation [ 76– 78] . These HSF1 condensates can compartmentalize Pol II and other transcription factors such as MED1, BRD4 and CycT1, enabling the rapid transcriptional induction of HSP genes. The increased HSP proteins also provide negative feedback to HSF1 condensates, preventing their solidification under extended heat-shock stress. Intriguingly, a recent study showed that the dissolution of foci coincides with the transcriptional activation of HSF1 target genes, leading to the proposal that HSF1 foci formation is inversely correlated with chaperone gene expression [76]. Although the foci may have a dominant-negative effect on gene activation by sequestering HSF1 from HSP promoters, other mechanisms also likely exist and require further investigation.

On the other hand, heat shock also leads to the suppression of global transcription, which is mainly caused by the disruption of Pol II′s progression beyond the initiation stage [ 79– 81] . It was recently discovered that NELFA, a subunit of the negative elongation factor (NELF) complex, forms nuclear condensates following heat shock stress. Importantly, these NELF condensates are distinct from HSF1 condensates and are formed in a phosphorylation and SUMOylation-dependent manner. Through phase separation, NELF condensates become stably associated with target genes, where they contribute to the decreased Pol II elongation and transcriptional suppression [82]. Therefore, both positive and negative condensates are enlisted by cells to orchestrate an efficient and specific transcriptional response to heat shock.

### Regulation of biomolecular condensates in response to DNA damage

Cells are constantly exposed to various types of genotoxic insults that cause DNA damage and genomic instability. These insults arise from a variety of sources, including exposure to radiation, chemotherapy drugs, as well as many endogenous metabolic side products. To cope with these threats, cells have developed a surveillance mechanism, known as DNA damage response (DDR), to promote DNA repair and protect genomic integrity [ 83, 84] . In addition to DDR, many other cellular processes, such as transcription, must be intricately regulated to enable the successful repair of DNA damage. On one hand, transcription can be silenced upon DNA damage, which is thought to prevent the accumulation of errors in mRNA and potentially the expression of abnormal proteins. On the other hand, transcription appears to promote DNA repair. For example, transcription-coupled DNA repair (TCR) is a well-characterized mechanism used by cells to rapidly remove DNA damage in actively transcribed genes. Therefore, transcription and DNA repair are two functionally intertwined processes.

Besides transcriptional condensates, many nuclear condensates formed via phase separation are also integral to the maintenance of genome integrity [ 85– 88] , but how these two types of functionally distinct condensates are coordinated in response to DNA damage is just beginning to emerge. Upon DNA double-strand breaks (DSBs) induction, a specific type of RNA known as damage-induced long non-coding RNAs (dilncRNAs) is rapidly synthesized at sites of DNA damage. These RNAs promote the assembly of DDR foci by driving the molecular crowding of DDR proteins into condensates [ 88, 89] . In this process, the induction of dilncRNA involves the formation of functional promoters near the DSBs, where Pol II, MED1, CDK9, and possibly other transcription factors are recruited to stimulate dilncRNA transcription. Therefore, it is likely that transcriptional condensates required for dilncRNA transcription are rapidly formed at the sites of damage, but more evidence is needed to confirm this.

Transcriptional condensates can also be dynamically modulated to promote transcriptional silencing in response to DNA damage. For example, CycT1 can concentrate Pol II and various transcription factors into a membraneless compartment to stimulate robust transcription elongation [54]. However, this elongation condensate is rapidly dissolved after DNA damage as a result of CycT1 PARylation by PARP1, leading to the inhibition of Pol II elongation [52]. Finally, condensates related to transcription may have harmful effects on genome integrity. A recent study found that active chromatins are trapped into condensates induced by transcriptional inhibition, where the frequency of illegitimate fusions of the DSBs is significantly increased, causing the formation of fusion oncogenes to promote tumorigenesis [90]. In light of these findings, it is conceivable that transcriptional condensates can have both positive and negative effects on DNA repair and are likely determined in a context-dependent manner.

### Regulation of biomolecular condensates in response to hyperosmotic stress

Hyperosmotic stress is a physiologic dysfunction that is caused by a sudden change in the solute concentration around a cell, leading to altered movement of water across the cell membrane. It usually occurs when cells are exposed to an environment that has a higher concentration of solutes than the cells themselves, and consequently, causing water loss and cell shrinkage [91]. Hyperosmotic stress is harmful to cells, as it can lead to cell death if it is not properly managed. Hence, cells have evolved a complex adaptive program to cope with the increased external osmolarity and protect cells from the damaging effects of osmotic shock.

Recent studies have provided novel insights into the role of transcriptional condensates in modulating rapid response to hyperosmotic stress. Adapting to osmotic stress is crucial for plant growth and crop yield, as it helps plants maintain proper hydration and ion balance in changing environments. SEU, a transcription regulator, is essential for plant growth and development [92], was recently reported to form phase-separated condensates and promote the expression of osmotic-related genes under hyperosmotic stress [93]. While the IDR of SEU is not necessary for normal plant growth, it is required for SEU condensation in response to hyperosmotic stress, suggesting that phase separation provides a means for the perception of osmotic stress in plants.

In mammalian cells, hyperosmotic stress has been shown to activate YAP signaling, which is a key effector of the Hippo pathway that activates various cellular functions [ 94, 95] . Like SEU, YAP can also form liquid-like condensates immediately following hyperosmotic stress [44]. These condensates shift the distribution of YAP from the cytoplasm to the nucleus, where it concentrates TEAD1 and many other transcription-related factors to drive the long-term expression of target genes. In addition to inducing transcriptional condensates, hyperosmotic stress also triggers rapid transcriptional changes in a broader context of gene expression control through other mechanisms. For example, the interactions between Pol II and Integrator complex subunits are dramatically decreased after salt stress, leading to widespread readthrough transcription and synthesis of downstream-of-gene-containing transcripts [96]. However, the role of phase separation in this process is yet to be understood.

### Biomolecular condensates related to signaling pathways

Signaling factors in the nucleus have been found to form biomolecular condensates to facilitate signal transductions in various cellular processes [97]. As discussed above, YAP is a downstream effector of the Hippo pathway and can form condensates to promote target gene expression in response to stress [44]. In addition to hyperosmotic stress, other upstream stimuli, such as IFN-γ signaling, have been shown to promote YAP condensation. These YAP-containing condensates were identified as transcription hubs to enhance the expression of key immunosuppressive target genes and the development of adaptive resistance in tumor cells to anti-PD-1 therapy [16]. Likewise, TAZ, the homolog of YAP, can also undergo phase separation to form liquid-like droplets. These TAZ condensates are highly dynamic and negatively regulated by phosphorylation mediated by LATS, enabling efficient and specific transcriptional activation of TAZ target genes [17].

Steroid hormones regulate various cellular processes through their effects on gene transcription, which involves the binding of hormones to their corresponding receptors and the coordination of genome-wide transcriptional programs. A growing body of evidence has suggested that phase separation extensively engages in hormone-activated gene expression, including that mediated by estrogen and androgen receptors. In breast cancer cells, acute activation of 17β-estradiol (E2) responsive enhancers requires the recruitment of a large protein complex, known as MegaTrans complex, in an estrogen receptor α (ERα) and enhancer RNA-dependent manner [98]. Recent studies suggested that these RNP assemblies exhibit properties of phase-separated condensates, which bring active enhancers into spatial proximity to facilitate their transcriptional coupling [46]. As with ER in breast cancer, androgen receptor (AR) is integral to the development and progression of prostate cancer. Recent studies showed that AR could undergo phase separation and form transcriptionally active condensates to stimulate oncogenic transcription programs in androgen-dependent prostate cancer cells [ 99, 100] . Notably, in a phase-separation-based phenotypic screen study, a lead compound specific for AR condensates disruption was developed, which shows effective inhibition of AR activity and proliferation even in cells expressing AR-resistant mutants [101]. Thus, this work offers a novel strategy to overcome drug resistance in prostate cancers.

In addition to the processes mentioned above, biomolecular condensates are involved in many other signaling pathways, including Wnt, TGF-β, and JAK/STAT [102]. As such, it is conceivable that many more signal pathway-related condensates are yet to be identified. Further efforts are required to determine the complete repertoire of these condensates and how they work to fine-tune the output of signal transduction.

## Transcriptional Condensates and Disease

**Figure 4:**
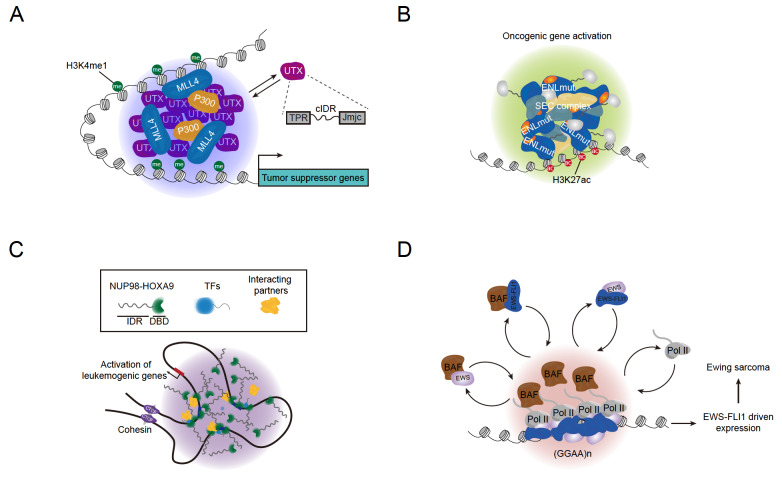
Figure 4 Dysregulation of transcriptional condensates and disease (A) The UTX protein contains an intrinsically disordered region that can contribute to the formation of transcriptional condensates, enabling the proper expression of tumor suppressor genes. However, mutations that disrupt the ability to form these condensates are commonly found in many human cancers, resulting in an inability to suppress tumor growth. (B) ENL mutants with mutated YEATS domains exhibit increased self-association, leading to an increased propensity to form biomolecular condensates that drive oncogenic gene activation. (C) NUP98-HOXA9 fusion forms phase-separated condensates to promote the transcriptional activation of leukemogenic genes. (D) In Ewing sarcoma, the EWS-FLI1 chimera concentrates the BAF complex and transcription machinery into liquid-like condensates at tumor-specific enhancers, contributing to the activation of target genes.

Cancer cells often exhibit altered gene expression patterns that support their uncontrolled proliferation and survival, which leads to the question of whether transcriptional condensates are hijacked by cancer cells to fulfill this purpose. Recent studies have provided important clues on how abnormal formation, composition, and altered biophysical properties of transcriptional condensates contribute to the development and progression of cancer ( Figure 4).

Ubiquitously transcribed tetratricopeptide repeat on chromosome X (UTX) is a specific histone H3K27 demethylase whose function is linked to diverse cellular processes [103]. A prominent feature of UTX is that it has tumor-suppressive activity and, accordingly, is frequently found mutated in human cancers. Interestingly, the demethylase activity of UTX does not appear to be a prerequisite for eliciting its tumor suppressive effects, leaving the exact mechanism underlying UTX-mediated tumor suppression unclear. Recent evidence suggests that UTX can undergo phase separation and recruit its key interaction partners, such as histone methyltransferase MLL4, to the same condensates [104]. This process is thought to promote efficient and correct histone modifications and high-order chromatin structures that are important for the proper expression of tumor suppressor genes activated by UTX. Notably, the phase separation ability of UTX can be attributed to a core intrinsically disordered region, which is deleted in some of the most frequent mutations of UTX in human cancers. This finding has potential clinical implications, as it suggests that rendering these UTX mutants with the ability to undergo phase separation may be a potential strategy for restoring their tumor suppressive activity. Although further investigations are needed to fully understand the role of UTX in cancer and the potential therapeutic benefits of restoring its phase separation, this study nonetheless establishes a crucial role for phase separation in UTX-mediated tumor suppression and showcases how the inability of forming biomolecular condensate due to genetic mutations can contribute to tumor development.

The eleven-nineteen-leukemia (ENL) is a chromatin reader that primarily recognizes H3K27ac through its YEATS domain. While the function of ENL in driving oncogenic gene expression programs has been well established, recent findings provided even deeper insights into the role of ENL and its phase separation in oncogenesis. The YEATS domain of ENL is a hotspot containing recurrent mutations found in many cancers, such as Wilms tumor and AML [105]. It was found that these oncogenic mutants exhibit enhanced propensity to undergo phase separation, leading to the formation of abnormal condensates in a dose-dependent manner. Mechanistically, ENL condensates are formed through multivalent homotypic and heterotypic interactions, with their perturbations resulting in the disruption of condensate formation and defects in oncogenic gene activation [106]. While these discoveries provide a framework for understanding the function of pathogenic condensates like those formed by ENL mutants, how such structures drive tumorigenesis in clinically-relevant animal models needs to be rigorously tested.

Other types of genetic alteration, especially those occurring in transcription factors, are also implicated in human pathologies associated with aberrant condensate formation. For instance, disease-associated repeat expansions in transcription factors have been shown to alter the biophysical properties of transcriptional condensates, which perturb the condensate composition and lead to transcriptional changes of target genes in disease-relevant cells [107]. In addition to repeat expansion, aberrant chimeras generated by chromosomal translocations of IDRs with chromatin-binding domains can also function as drivers in human malignancies. In the case of leukemia driven by recurrent NUP98-HOXA9 translocation, the chimera can form phase-separated puncta in the nucleus, where it promotes the occupancy of chimera transcription factors and the transcriptional activation of leukemogenic genes [ 108, 109] . Similarly, condensates formed by EWS-FLI1 chimera have also been reported to drive the oncogenic transformation and development of Ewing sarcoma [ 110, 111] . These findings suggest that multiple mechanisms exist to initiate the formation of dysregulated transcriptional condensates by disease-causing transcription factors, which play a critical role in the development and progression of cancer.

## Concluding Remarks

In this review, we have mainly focused on the role of phase separation in Pol II-mediated transcription, ranging from the basal transcriptional condensates that are involved in regulating each stage of the transcription cycle to the conditional transcriptional condensates that are swiftly formed and regulated to enable rapid transcriptional response. We also highlight the fundamental principles and molecular mechanisms that underlie the dynamical changes in these transcriptional condensates and how their dysregulation is linked to many human diseases, pointing therapeutic strategies that target transcriptional condensates as potential treatments for these diseases.

Despite the rapid progress in the field of phase separation since its first description in 2009, many knowledge gaps remain to be filled to fully understand the role of phase separation in transcription control. For example, transcriptional condensates involved in transcription initiation and elongation have been well-studied, however, whether or not phase separation contributes to other aspects of transcription, such as termination, remains unclear. Given that transcription termination requires coordinated actions of myriads of factors, it is likely that phase separation is also involved in this process. Nevertheless, future studies are required to experimentally confirm this point.

Defining the composition of transcriptional condensate is another outstanding question that needs to be addressed. While key drivers are important for transcriptional condensate formation, other components present in transcriptional condensate are also essential for transcriptional regulation. These “client” proteins can provide multiple layers of regulation on condensate formation, dynamics, and dissolution, therefore greatly expanding the functional flexibility and diversity of transcriptional condensates for gene expression control. However, compared to the drivers of transcriptional condensate that have been extensively studied, we know surprisingly little about client proteins. This is partly due to the lack of appropriate approaches to faithfully capture weak and multivalent interactions within phase-separated compartments. As such, innovative purification and proteomic strategies capable of mapping the composition of phase-separated condensates are urgently needed. The development of such approaches will provide invaluable tools for gaining a better appreciation of transcriptional control by phase separation mechanism.

Finally, although the concept of phase separation has transformed our understanding of gene transcription control, there are still controversies regarding the biological functions of transcriptional condensates. For example, further study is needed to comprehensively define the functional importance of transcriptional condensates at the organism level. The role of phase separation in transcriptional activation should also be rigorously explored, as recent findings argue that phase separation has no effect or may even suppress transcription [ 111, 112] . In this regard, advances in the development of microscopic technology with a higher resolution as well as systems to study transcription at endogenous genomic loci will provide significant momentum for the field to assess the functional role of transcriptional condensates for gene activation. Furthermore, how the spatiotemporal partitioning of various transcriptional regulators into specific transcriptional condensates is established remains an outstanding question [62]. On the basis of a recent study, both the disordered regions and charge patterning of transcriptional regulators are required for the selective compartmentalization of functionally related proteins for transcriptional activation [113]. However, whether other mechanisms are also involved in the selective partitioning of transcriptional regulators to assemble condensates needs to be explored. While challenges remain in this rapidly growing field, many exciting and novel discoveries and concepts will likely continue to emerge and propel the field further to unravel the complex mechanisms of transcriptional regulation.
